# Supplementary material for: Complexome profiling of the Chlamydomonas psb28 mutant reveals TEF5 as an early PSII assembly factor
Source: Plant Cell. 2025 Mar 17;37(6):koaf055. doi: 10.1093/plcell/koaf055 (PMC12164590; doi:10.1093/plcell/koaf055)
Supplement: koaf055_Supplementary_Data [file koaf055_supplementary_data.zip › psb28_interactive.zip!/plots/intensity/qualityPlots/peptides/_Cre02.g142266.t1.1.html]

### Cre02.g142266.t1.1

Trivial name:   
SeqCoverage [median]: 5.40  
Mass: 71.73 kDa  
  
 

| Sequence | MissedCleavage | Mass [Da] | wtIntensity | muIntensity | Score |
| --- | --- | --- | --- | --- | --- |
| LGENLEDR | 0 | 944.46 | 776.32 | 356.24 | 83.21 |
| LGLDIIGK | 0 | 827.51 | 1082.05 | 0.00 | 77.06 |
|  |  |  | 1858.4 | 356.2 |  |
